# The Contribution of Genetics to Muscle Disuse, Retraining, and Aging

**DOI:** 10.3390/genes13081378

**Published:** 2022-08-01

**Authors:** Giuseppe Sirago, Anna Picca, Emiliana Giacomello, Emanuele Marzetti, Luana Toniolo

**Affiliations:** 1 Department of Biomedical Sciences, University of Padova, 35131 Padova, Italy; luana.toniolo@unipd.it; 2 Fondazione Policlinico Universitario “Agostino Gemelli” IRCCS, 00168 Roma, Italy; anna.picca@policlinicogemelli.it (A.P.); emanuele.marzetti@policlinicogemelli.it (E.M.); 3 Department of Medicine, Surgery and Health Sciences, University of Trieste, 34127 Trieste, Italy; egiacomello@units.it; 4 Department of Geriatrics and Orthopedics, Università Cattolica del Sacro Cuore, 00168 Roma, Italy

**Keywords:** muscle atrophy, exercise, genetic variants

## Abstract

Genetic background may partly explain differences in muscle responses to internal or external stimuli. Muscle disuse involves various degrees of skeletal muscle atrophy due to inactivity and mechanical unloading. Whether and to which extent genetic background impacts disuse atrophy and retraining in individuals of different ages are currently unclear. Here, we provide a brief overview of relevant literature on the contribution of genetics to muscle disuse, retraining, and aging, and offer a perspective on unanswered questions on the subject that may open new venues for research.

## 1. Skeletal Muscle Plasticity and Genetic Variants

Disuse and physical exercise are two opposite ‘insults’ to the skeletal muscle that induce phenotypic changes and adaptations [1], with substantial inter-individual variability [2,3]. Muscle disuse causes loss of sarcomere myoproteins leading to activation of gene atrophy programs and muscle protein degradation [1,4]. Conversely, exercise stimulates muscle protein synthesis with deposition of new structural proteins, ultimately resulting in muscle fibre accretion [1]. At the systemic level, exercise induces prompt release of inflammatory molecules (e.g., interleukin 6), followed by secretion of anti-inflammatory mediators aimed at counteracting a hyperactive inflammatory state [5]. Little data are available on the role of inflammation in disuse muscle atrophy, although neuromuscular instability and oxidative stress have been indicated as early events following atrophic insults.

The molecular mechanisms involved in disuse muscle atrophy vary depending on the duration of muscle unloading. In humans and animal models, the major events that trigger the activation of the atrophy gene program seem to occur during the first 5–10 days of muscle unloading [6,7,8,9,10]. In rodents, transcriptional changes of regulatory genes occur on day 1 and continue until day 4 of disuse [10]. These events precede the downregulation of genes coding for sarcomere proteins and the upregulation of atrogenes, resulting in disassembly of myofibrillar proteins from the sarcomere and their subsequent degradation [10].

Differences in genetic backgrounds may affect the response to atrophying stimuli and partly explain phenotypic heterogeneity of disuse muscle atrophy. Table 1 lists genetic variants associated with muscle atrophy due to inactivity, post-disuse muscle recovery upon retraining, and aging. Special focus is placed on genetic variants that may shed light on the molecular mechanisms involved in muscle adaptations to varying loading conditions and support the development of therapeutic strategies to counteract muscle atrophy.

The literature search revealed that the influence of genetics in developing disuse and in mounting the response to retraining has been sparsely investigated. In addition, most studies are associative and a substantial gap in knowledge remains as to whether genetic variants indeed have an influence on the mechanisms contributing to muscle disuse, retraining, and aging.

Only two studies investigated the influence of genetic background on muscle mass loss or regain upon retraining in murine models [11,12]. No such studies have been conducted in humans. In mice, muscle responses to disuse differ according to strains. Depending on the genetic background, muscle atrophy induced by cast immobilization was found to be mostly driven either by upregulation of atrogenes expression or downregulation of protein synthesis [12]. However, variations in genetic makeup account for only 5% of inter-individual variability in the extent of disuse muscle atrophy [11], which indicates that other factors (e.g., epigenetic modulations) might be more relevant in determining muscle responses to immobilization.

Besides structural alterations, disuse causes substantial metabolic changes in muscle, such as insulin resistance. Short-term disuse was shown to induce various degrees of insulin resistance in middle-aged healthy volunteers [13]. In particular, transcriptomic analyses showed that those whose insulin sensitivity decreased to the largest extent displayed greater downregulation of muscle genes involved in lipid uptake and oxidation, export of triglyceride, lipogenesis, and amino acid export [13].

Studies have indicated a role for SNP variants in muscle performance of athletes, but less is known on the matter in community-dwelling people or during aging. Vitamin D receptor genotypes have been linked with differences in quadriceps and handgrip strength among non-obese women [20]. Myostatin is a negative regulator of muscle mass that is counterbalanced by follistatin. The MSTN K153R polymorphism has been indicated as the so-called ‘explosive’ leg power in non-athlete men [13]. Moreover, myostatin polymorphisms along with SNPs in follistatin have been associated with inter-individual variability in muscle strength in African Americans [19]. Myostatin and follistatin gene variants are also linked to susceptibility to age-associated muscle mass and strength declines [14,15,18,19]. In addition, structural genes involved in microtubule and trabecular skeletal muscle structure, such as actinin-3, seem to have a role in attenuating muscle atrophy during aging [16]. Finally, an association between SNPs in regulatory regions and measures of physical performance has been described in older adults [21]. Further studies are needed, especially in humans, to confirm these initial findings.

Finally, statistical tools that account for the individual genetic background as a system responding to specific stimuli are needed to understand how genetics may influence the response of muscle to various injuries. This may allow for developing approaches to support the selection of an ad-hoc population to conduct longitudinal studies.

## 2. Conclusions

In the present perspective, we have briefly discussed relevant literature on muscle responses to disuse, retraining, and aging in humans and animal models. The output of our literature search highlights the need for additional studies that may help clarify the contribution of specific genetic variants to the mechanisms underlying muscle disuse, post-disuse recovery, and aging. We have also suggested the opportunity of developing statistical approaches that may help capture the overall genetic background as a system that responds to a stimulus. This holistic approach may help address unanswered questions in the field of muscle adaptations to loading conditions and aging, and may open new research venues.

## Figures and Tables

**Table 1:** Genetics variants associated with muscle disuse, retraining, and aging.

Genetic Variant	Variability	Species	Reference
QTL on Chr 5	Loss in CSA upon disuse	Mouse	Judex et al., 2016 [11]
QTL on Chr 2 & 19	Gain in CSA upon retraining	Mouse	Judex et al., 2016 [11]
129S1/SvlmJ strain	Resistance to muscle loss	Mouse	Maroni et al., 2021 [12]
NOD/ShiLtJ & NZO/HILtJ strains	Susceptibility to muscle loss	Mouse	Maroni et al., 2021 [12]
CAST/EiJ strain	Compensation between MPB and MPS upon disuse	Mouse	Maroni et al., 2021 [12]
PFKFB3, FASN & SLC43A1	Insulin resistance upon disuse	Human	Mahmassani et al., 2019 [13]
A55T & K153R MSTN	Muscle power and hypertrophy in non-athletes	Human	Santiago et al., 2011 [14]; Li et al., 2014 [15]
ACTN3 R577X	Muscle power with aging	Human	Delmonico et al., 2008 [16]
CNTFR C1703T & T1069A	Muscle strength with aging	Human	De Mars et al., 2007 [17]
ACVR2B and FSTL	Muscle mass and strength with aging	Human	Walsh et al., 2007 [18]
MSTN A2379G & FST A5003T	Muscle strength and size in young African Americans	Human	Kostek et al., 2009 [19]
bb VDR	Quadriceps strength in non-obese women	Human	Geusens et al., 1997 [20]
ZNF295 & C2CD2	Muscle function in aging	Human	Heckerman et al., 2017 [21]

Abbreviations: CSA, cross-sectional area; MPB, muscle protein breakdown; MPS: muscle protein synthesis.

## Data Availability

Not applicable.
